# Apoptosis in cancer: from pathogenesis to treatment

**DOI:** 10.1186/1756-9966-30-87

**Published:** 2011-09-26

**Authors:** Rebecca SY Wong

**Affiliations:** 1 Division of Human Biology, School of Medical and Health Sciences, International Medical University. No. 126, Jalan Jalil Perkasa 19, Bukit Jalil 57000 Kuala Lumpur, Malaysia

**Keywords:** Apoptosis, defective apoptotic pathways, carcinogenesis, treatment target

## Abstract

Apoptosis is an ordered and orchestrated cellular process that occurs in physiological and pathological conditions. It is also one of the most studied topics among cell biologists. An understanding of the underlying mechanism of apoptosis is important as it plays a pivotal role in the pathogenesis of many diseases. In some, the problem is due to too much apoptosis, such as in the case of degenerative diseases while in others, too little apoptosis is the culprit. Cancer is one of the scenarios where too little apoptosis occurs, resulting in malignant cells that will not die. The mechanism of apoptosis is complex and involves many pathways. Defects can occur at any point along these pathways, leading to malignant transformation of the affected cells, tumour metastasis and resistance to anticancer drugs. Despite being the cause of problem, apoptosis plays an important role in the treatment of cancer as it is a popular target of many treatment strategies. The abundance of literature suggests that targeting apoptosis in cancer is feasible. However, many troubling questions arise with the use of new drugs or treatment strategies that are designed to enhance apoptosis and critical tests must be passed before they can be used safely in human subjects.

## 1. Introduction

Cell death, particularly apoptosis, is probably one of the most widely-studied subjects among cell biologists. Understanding apoptosis in disease conditions is very important as it not only gives insights into the pathogenesis of a disease but may also leaves clues on how the disease can be treated. In cancer, there is a loss of balance between cell division and cell death and cells that should have died did not receive the signals to do so. The problem can arise in any one step along the way of apoptosis. One example is the downregulation of p53, a tumour suppressor gene, which results in reduced apoptosis and enhanced tumour growth and development [1] and inactivation of p53, regardless of the mechanism, has been linked to many human cancers [2-4]. However, being a double-edged sword, apoptosis can be cause of the problem as well as the solution, as many have now ventured into the quest of new drugs targeting various aspects of apoptosis [5,6]. Hence, apoptosis plays an important role in both carcinogenesis and cancer treatment. This article gives a comprehensive review of apoptosis, its mechanisms, how defects along the apoptotic pathway contribute to carcinogenesis and how apoptosis can be used as a vehicle of targeted treatment in cancer.

## 2. Apoptosis

The term "apoptosis" is derived from the Greek words "*απο*" and "*πτωσιζ*" meaning "dropping off" and refers to the falling of leaves from trees in autumn. It is used, in contrast to necrosis, to describe the situation in which a cell actively pursues a course toward death upon receiving certain stimuli [7]. Ever since apoptosis was described by Kerr *et al *in the 1970's, it remains one of the most investigated processes in biologic research [8]. Being a highly selective process, apoptosis is important in both physiological and pathological conditions [9,10]. These conditions are summarised in Table 1.

**Table 1:** Conditions involving apoptosis

**Physiological conditions**
Programmed cell destruction in embryonic development for the purpose of sculpting of tissue
Physiologic involution such as shedding of the endometrium, regression of the lactating breast
Normal destruction of cells accompanied by replacement proliferation such as in the gut epithelium
Involution of the thymus in early age
**Pathological conditions**
Anticancer drug induced cell death in tumours
Cytotoxic T cell induced cell death such as in immune rejection and graft versus host disease
Progressive cell death and depletion of CD4+ cells in AIDs
Some forms of virus-induced cell death, such as hepatitis B or C
Pathologic atrophy of organs and tissues as a result of stimuli removal e.g. prostatic atrophy after orchidectomy
Cell death due to injurious agents like radiation, hypoxia and mild thermal injury
Cell death in degenerative diseases such as Alzheimer's disease and Parkinson's disease
Cell death that occurs in heart diseases such as myocardial infarction

### 2.1 Morphological changes in apoptosis

Morphological alterations of apoptotic cell death that concern both the nucleus and the cytoplasm are remarkably similar across cell types and species [11,12]. Usually several hours are required from the initiation of cell death to the final cellular fragmentation. However, the time taken depends on the cell type, the stimulus and the apoptotic pathway [13].

Morphological hallmarks of apoptosis in the nucleus are chromatin condensation and nuclear fragmentation, which are accompanied by rounding up of the cell, reduction in cellular volume (pyknosis) and retraction of pseudopodes [14]. Chromatin condensation starts at the periphery of the nuclear membrane, forming a crescent or ring-like structure. The chromatin further condenses until it breaks up inside a cell with an intact membrane, a feature described as karyorrhexis [15]. The plasma membrane is intact throughout the total process. At the later stage of apoptosis some of the morphological features include membrane blebbing, ultrastrutural modification of cytoplasmic organelles and a loss of membrane integrity [14]. Usually phagocytic cells engulf apoptotic cells before apoptotic bodies occur. This is the reason why apoptosis was discovered very late in the history of cell biology in 1972 and apoptotic bodies are seen *in vitro *under special conditions. If the remnants of apoptotic cells are not phagocytosed such as in the case of an artificial cell culture environment, they will undergo degradation that resembles necrosis and the condition is termed secondary necrosis [13].

### 2.2 Biochemical changes in apoptosis

Broadly, three main types of biochemical changes can be observed in apoptosis: 1) activation of caspases, 2) DNA and protein breakdown and 3) membrane changes and recognition by phagocytic cells [16]. Early in apoptosis, there is expression of phosphatidylserine (PS) in the outer layers of the cell membrane, which has been "flipped out" from the inner layers. This allows early recognition of dead cells by macrophages, resulting in phagocytosis without the release of pro-inflammatory cellular components [17]. This is followed by a characteristic breakdown of DNA into large 50 to 300 kilobase pieces [18]. Later, there is internucleosomal cleavage of DNA into oligonucleosomes in multiples of 180 to 200 base pairs by endonucleases. Although this feature is characteristic of apoptosis, it is not specific as the typical DNA ladder in agarose gel electrophoresis can be seen in necrotic cells as well [19]. Another specific feature of apoptosis is the activation of a group of enzymes belonging to the cysteine protease family named caspases. The "c" of "caspase" refers to a cysteine protease, while the "aspase" refers to the enzyme's unique property to cleave after aspartic acid residues [16]. Activated caspases cleave many vital cellular proteins and break up the nuclear scaffold and cytoskeleton. They also activate DNAase, which further degrade nuclear DNA [20]. Although the biochemical changes explain in part some of the morphological changes in apoptosis, it is important to note that biochemical analyses of DNA fragmentation or caspase activation should not be used to define apoptosis, as apoptosis can occur without oligonucleosomal DNA fragmentation and can be caspase-independent [21]. While many biochemical assays and experiments have been used in the detection of apoptosis, the Nomenclature Committee on Cell Death (NCCD) has proposed that the classification of cell death modalities should rely purely on morphological criteria because there is no clear-cut equivalence between ultrastructural changes and biochemical cell death characteristics [21].

### 2.3 Mechanisms of apoptosis

**Figure 1:**
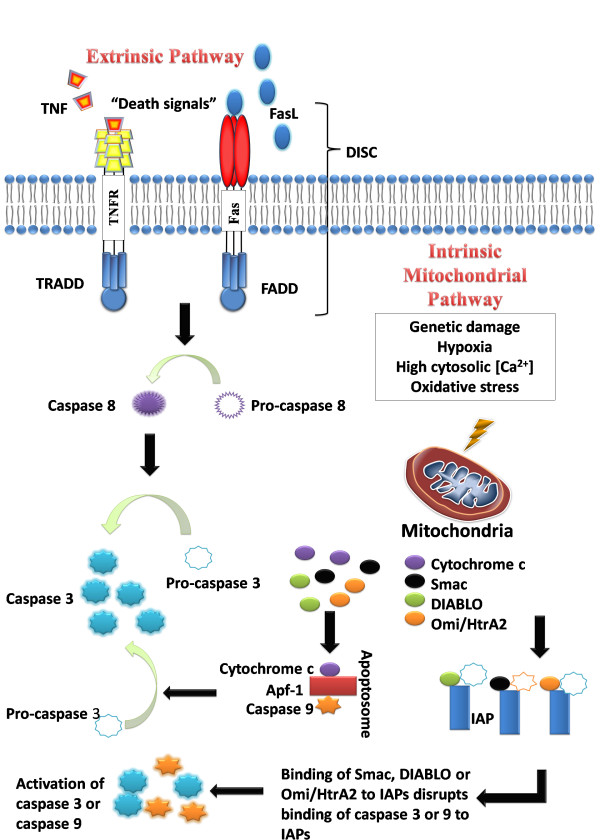
**The intrinsic and extrinsic pathways of apoptosis**.

Understanding the mechanisms of apoptosis is crucial and helps in the understanding of the pathogenesis of conditions as a result of disordered apoptosis. This in turn, may help in the development of drugs that target certain apoptotic genes or pathways. Caspases are central to the mechanism of apoptosis as they are both the initiators and executioners. There are three pathways by which caspases can be activated. The two commonly described initiation pathways are the intrinsic (or mitochondrial) and extrinsic (or death receptor) pathways of apoptosis (Figure 1). Both pathways eventually lead to a common pathway or the execution phase of apoptosis. A third less well-known initiation pathway is the intrinsic endoplasmic reticulum pathway [22].

#### 2.3.1 The extrinsic death receptor pathway

The extrinsic death receptor pathway, as its name implies, begins when death ligands bind to a death receptor. Although several death receptors have been described, the best known death receptors is the type 1 TNF receptor (TNFR1) and a related protein called Fas (CD95) and their ligands are called TNF and Fas ligand (FasL) respectively [17]. These death receptors have an intracellular death domain that recruits adapter proteins such as TNF receptor-associated death domain (TRADD) and Fas-associated death domain (FADD), as well as cysteine proteases like caspase 8 [23]. Binding of the death ligand to the death receptor results in the formation of a binding site for an adaptor protein and the whole ligand-receptor-adaptor protein complex is known as the death-inducing signalling complex (DISC) [22]. DISC then initiates the assembly and activation of pro-caspase 8. The activated form of the enzyme, caspase 8 is an initiator caspase, which initiates apoptosis by cleaving other downstream or executioner caspases [24].

#### 2.3.2 The intrinsic mitochondrial pathway

As its name implies, the intrinsic pathway is initiated within the cell. Internal stimuli such as irreparable genetic damage, hypoxia, extremely high concentrations of cytosolic Ca^2+ ^and severe oxidative stress are some triggers of the initiation of the intrinsic mitochondrial pathway [24]. Regardless of the stimuli, this pathway is the result of increased mitochondrial permeability and the release of pro-apoptotic molecules such as cytochrome-c into the cytoplasm [25]. This pathway is closely regulated by a group of proteins belonging to the Bcl-2 family, named after the BCL2 gene originally observed at the chromosomal breakpoint of the translocation of chromosome 18 to 14 in follicular non-Hodgkin lymphoma [26]. There are two main groups of the Bcl-2 proteins, namely the pro-apoptotic proteins (e.g. Bax, Bak, Bad, Bcl-Xs, Bid, Bik, Bim and Hrk) and the anti-apoptotic proteins (e.g. Bcl-2, Bcl-X_L_, Bcl-W, Bfl-1 and Mcl-1) [27]. While the anti-apoptotic proteins regulate apoptosis by blocking the mitochondrial release of cytochrome-c, the pro-apoptotic proteins act by promoting such release. It is not the absolute quantity but rather the balance between the pro- and anti-apoptotic proteins that determines whether apoptosis would be initiated [27]. Other apoptotic factors that are released from the mitochondrial intermembrane space into the cytoplasm include apoptosis inducing factor (AIF), second mitochondria-derived activator of caspase (Smac), direct IAP Binding protein with Low pI (DIABLO) and Omi/high temperature requirement protein A (HtrA2) [28]. Cytoplasmic release of cytochrome c activates caspase 3 via the formation of a complex known as apoptosome which is made up of cytochrome c, Apaf-1 and caspase 9 [28]. On the other hand, Smac/DIABLO or Omi/HtrA2 promotes caspase activation by binding to inhibitor of apoptosis proteins (IAPs) which subsequently leads to disruption in the interaction of IAPs with caspase-3 or -9 [28,29].

#### 2.3.3 The common pathway

The execution phase of apoptosis involves the activation of a series of caspases. The upstream caspase for the intrinsic pathway is caspase 9 while that of the extrinsic pathway is caspase 8. The intrinsic and extrinsic pathways converge to caspase 3. Caspase 3 then cleaves the inhibitor of the caspase-activated deoxyribonuclease, which is responsible for nuclear apoptosis [30]. In addition, downstream caspases induce cleavage of protein kinases, cytoskeletal proteins, DNA repair proteins and inhibitory subunits of endonucleases family. They also have an effect on the cytoskeleton, cell cycle and signalling pathways, which together contribute to the typical morphological changes in apoptosis [30].

#### 2.3.4 The intrinsic endoplasmic reticulum pathway

This intrinsic endoplasmic reticulum (ER) pathway is a third pathway and is less well known. It is believed to be caspase 12-dependent and mitochondria-independent [31]. When the ER is injured by cellular stresses like hypoxia, free radicals or glucose starvation, there is unfolding of proteins and reduced protein synthesis in the cell, and an adaptor protein known as TNF receptor associated factor 2 (TRAF2) dissociates from procaspase-12, resulting in the activation of the latter [22].

## 3. Apoptosis and carcinogenesis

**Figure 2:**
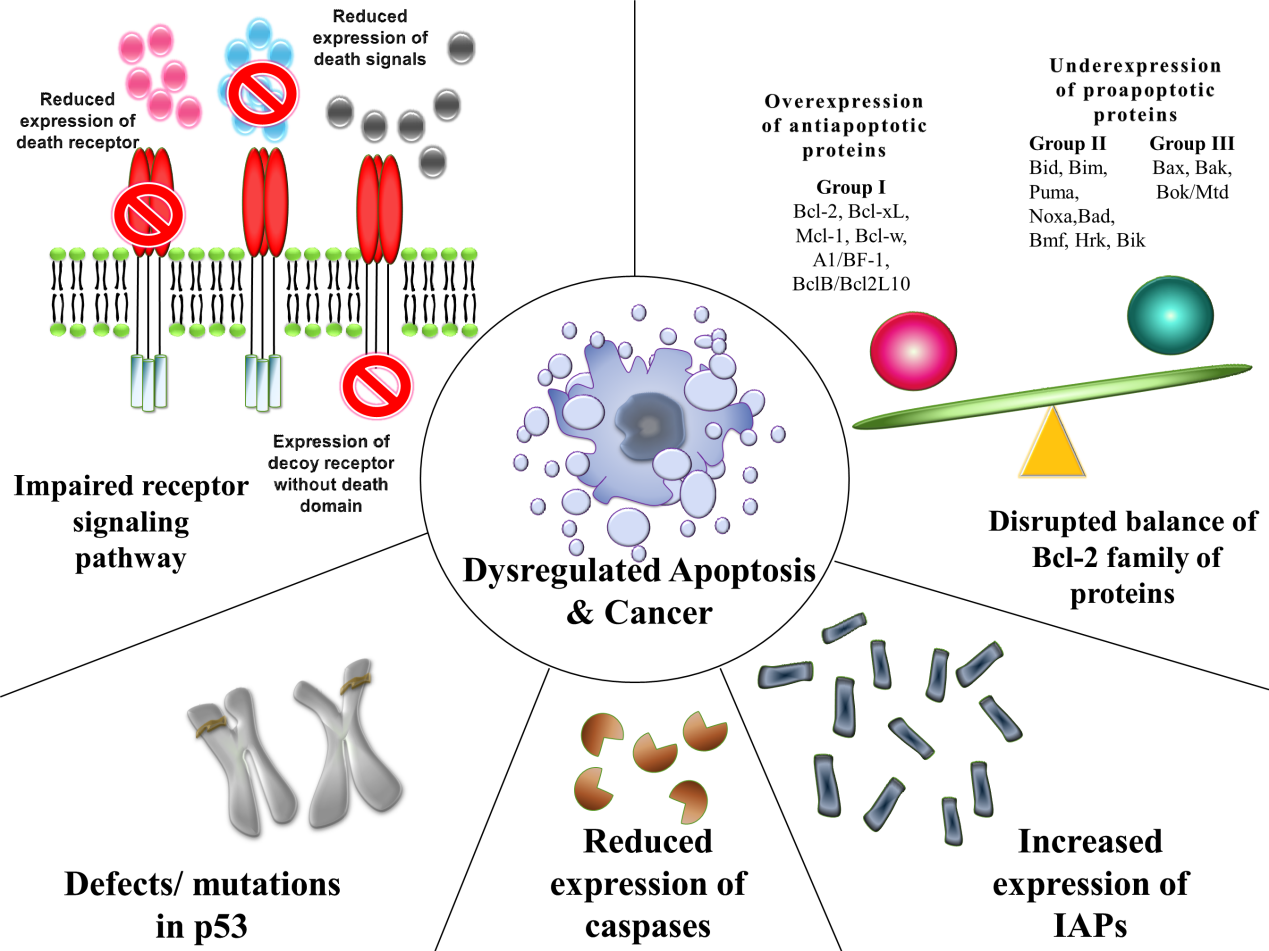
**Mechanisms contributing to evasion of apoptosis and carcinogenesis**.

Cancer can be viewed as the result of a succession of genetic changes during which a normal cell is transformed into a malignant one while evasion of cell death is one of the essential changes in a cell that cause this malignant transformation [32]. As early as the 1970's, Kerr *et al *had linked apoptosis to the elimination of potentially malignant cells, hyperplasia and tumour progression [8]. Hence, reduced apoptosis or its resistance plays a vital role in carcinogenesis. There are many ways a malignant cell can acquire reduction in apoptosis or apoptosis resistance. Generally, the mechanisms by which evasion of apoptosis occurs can be broadly dividend into: 1) disrupted balance of pro-apoptotic and anti-apoptotic proteins, 2) reduced caspase function and 3) impaired death receptor signalling. Figure 2 summarises the mechanisms that contribute to evasion of apoptosis and carcinogenesis.

### 3.1 Disrupted balance of pro-apoptotic and anti-apoptotic proteins

Many proteins have been reported to exert pro- or anti-apoptotic activity in the cell. It is not the absolute quantity but rather the ratio of these pro-and anti-apoptotic proteins that plays an important role in the regulation of cell death. Besides, over- or under-expression of certain genes (hence the resultant regulatory proteins) have been found to contribute to carcinogenesis by reducing apoptosis in cancer cells.

#### 3.1.1 The Bcl-2 family of proteins

The Bcl-2 family of proteins is comprised of pro-apoptotic and anti-apoptotic proteins that play a pivotal role in the regulation of apoptosis, especially via the intrinsic pathway as they reside upstream of irreversible cellular damage and act mainly at the mitochondria level [33]. Bcl-2 was the first protein of this family to be identified more than 20 years ago and it is encoded by the BCL2 gene, which derives its name from B-cell lymphoma 2, the second member of a range of proteins found in human B-cell lymphomas with the t (14; 18) chromosomal translocation [26].

All the Bcl-2 members are located on the outer mitochondrial membrane. They are dimmers which are responsible for membrane permeability either in the form of an ion channel or through the creation of pores [34]. Based of their function and the Bcl-2 homology (BH) domains the Bcl-2 family members are further divided into three groups [35]. The first group are the anti-apoptotic proteins that contain all four BH domains and they protect the cell from apoptotic stimuli. Some examples are Bcl-2, Bcl-xL, Mcl-1, Bcl-w, A1/Bfl-1, and Bcl-B/Bcl2L10. The second group is made up of the BH-3 only proteins, so named because in comparison to the other members, they are restricted to the BH3 domain. Examples in this group include Bid, Bim, Puma, Noxa, Bad, Bmf, Hrk, and Bik. In times of cellular stresses such as DNA damage, growth factor deprivation and endoplasmic reticulum stress, the BH3-only proteins, which are initiators of apoptosis, are activated. Therefore, they are pro-apoptotic. Members of the third group contain all four BH domains and they are also pro-apoptotic. Some examples include Bax, Bak, and Bok/Mtd [35].

When there is disruption in the balance of anti-apoptotic and pro-apoptotic members of the Bcl-2 family, the result is dysregulated apoptosis in the affected cells. This can be due to an overexpression of one or more anti-apoptotic proteins or an underexpression of one or more pro-apoptotic proteins or a combination of both. For example, Raffo *et al *showed that the overexpression of Bcl-2 protected prostate cancer cells from apoptosis [36] while Fulda *et al *reported Bcl-2 overexpression led to inhibition of TRAIL-induced apoptosis in neuroblastoma, glioblastoma and breast carcinoma cells [37]. Overexpression of Bcl-xL has also been reported to confer a multi-drug resistance phenotype in tumour cells and prevent them from undergoing apoptosis [38]. In colorectal cancers with microsatellite instability, on the other hand, mutations in the *bax *gene are very common. Miquel *et al *demonstrated that impaired apoptosis resulting from *bax*(G)8 frameshift mutations could contribute to resistance of colorectal cancer cells to anticancer treatments [39]. In the case of chronic lymphocytic leukaemia (CLL), the malignant cells have an anti-apoptotic phenotype with high levels of anti-apoptotic Bcl-2 and low levels of pro-apoptotic proteins such as Bax *in vivo*. Leukaemogenesis in CLL is due to reduced apoptosis rather than increased proliferation *in vivo *[40]. Pepper *et al *reported that B-lymphocytes in CLL showed an increased Bcl-2/Bax ratio in patients with CLL and that when these cells were cultured *in vitro*, drug-induced apoptosis in B-CLL cells was inversely related to Bcl-2/Bax ratios [41].

#### 3.1.2 p53

The p53 protein, also called tumour protein 53 (or TP 53), is one of the best known tumour suppressor proteins encoded by the tumour suppressor gene *TP53 *located at the short arm of chromosome 17 (17p13.1). It is named after its molecular weights, i.e., 53 kDa [42]. It was first identified in 1979 as a transformation-related protein and a cellular protein accumulated in the nuclei of cancer cells binding tightly to the simian virus 40 (SV40) large T antigen. Initially, it was found to be weakly-oncogenic. It was later discovered that the oncogenic property was due to a p53 mutation, or what was later called a "gain of oncogenic function" [43]. Since its discovery, many studies have looked into its function and its role in cancer. It is not only involved in the induction of apoptosis but it is also a key player in cell cycle regulation, development, differentiation, gene amplification, DNA recombination, chromosomal segregation and cellular senescence [44] and is called the "guardian of the genome" [45].

Defects in the p53 tumour suppressor gene have been linked to more than 50% of human cancers [43]. Recently, Avery-Kieida *et al *reported that some target genes of p53 involved in apoptosis and cell cycle regulation are aberrantly expressed in melanoma cells, leading to abnormal activity of p53 and contributing to the proliferation of these cells [46]. In a mouse model with an *N*-terminal deletion mutant of p53 (Δ122p53) that corresponds to Δ133p53, Slatter *et al *demonstrated that these mice had decreased survival, a different and more aggressive tumor spectrum, a marked proliferative advantage on cells, reduced apoptosis and a profound proinflammatory phenotype [47]. In addition, it has been found that when the p53 mutant was silenced, such down-regulation of mutant p53 expression resulted in reduced cellular colony growth in human cancer cells, which was found to be due to the induction of apoptosis [48].

#### 3.1.3 Inhibitor of apoptosis proteins (IAPs)

The inhibitor of apoptosis proteins are a group of structurally and functionally similar proteins that regulate apoptosis, cytokinesis and signal transduction. They are characterised by the presence of a baculovirus IAP repeat (BIR) protein domain [29]. To date eight IAPs have been identified, namely, NAIP (BIRC1), c-IAP1 (BIRC2), c-IAP2 (BIRC3), X-linked IAP (XIAP, BIRC4), Survivin (BIRC5), Apollon (BRUCE, BIRC6), Livin/ML-IAP (BIRC7) and IAP-like protein 2 (BIRC8) [49]. IAPs are endogenous inhibitors of caspases and they can inhibit caspase activity by binding their conserved BIR domains to the active sites of caspases, by promoting degradation of active caspases or by keeping the caspases away from their substrates [50].

Dysregulated IAP expression has been reported in many cancers. For example, Lopes *et al *demonstrated abnormal expression of the IAP family in pancreatic cancer cells and that this abnormal expression was also responsible for resistance to chemotherapy. Among the IAPs tested, the study concluded that drug resistance correlated most significantly with the expression of cIAP-2 in pancreatic cells [51]. On the other hand, Livin was demonstrated to be highly expressed in melanoma and lymphoma [52,53] while Apollon, was found to be upregulated in gliomas and was responsible for cisplatin and camptothecin resistance [54]. Another IAP, Survivin, has been reported to be overexpressed in various cancers. Small *et al*. observed that transgenic mice that overexpressed Survivin in haematopoietic cells were at an increased risk of haematological malignancies and that haematopoietic cells engineered to overexpress Survivin were less susceptible to apoptosis [55]. Survivin, together with XIAP, was also found to be overexpressed in non-small cell lung carcinomas (NSCLCs) and the study concluded that the overexpression of Survivin in the majority of NSCLCs together with the abundant or upregulated expression of XIAP suggested that these tumours were endowed with resistance against a variety of apoptosis-inducing conditions [56].

### 3.2 Reduced capsase activity

The caspases can be broadly classified into two groups: 1) those related to caspase 1 (e.g. caspase-1, -4, -5, -13, and -14) and are mainly involved in cytokine processing during inflammatory processes and 2) those that play a central role in apoptosis (e.g. caspase-2, -3. -6, -7,-8, -9 and -10). The second group can be further classified into 1) initiator caspases (e.g. caspase-2, -8, -9 and -10) which are primarily responsible for the initiation of the apoptotic pathway and 2) effector caspases (caspase-3, -6 and -7) which are responsible in the actual cleavage of cellular components during apoptosis [57]. As mentioned in Section 2.2, caspases remain one of the important players in the initiation and execution of apoptosis. It is therefore reasonable to believe that low levels of caspases or impairment in caspase function may lead to a decreased in apoptosis and carcinogenesis.

In one study, downregulation of caspase-9 was found to be a frequent event in patients with stage II colorectal cancer and correlates with poor clinical outcome [58]. In another study, Devarajan *et al *observed that caspases-3 mRNA levels in commercially available total RNA samples from breast, ovarian, and cervical tumuors were either undetectable (breast and cervical) or substantially decreased (ovarian) and that the sensitivity of caspase-3-deficient breast cancer (MCF-7) cells to undergo apoptosis in response to anticancer drug or other stimuli of apoptosis could be enhanced by restoring caspase-3 expression, suggesting that the loss of caspases-3 expression and function could contribute to breast cancer cell survival [59]. In some instances, more than one caspase can be downregulated, contributing to tumour cell growth and development. In a cDNA array differential expression study, Fong *et al *observed a co-downregulation of both capase-8 and -10 and postulated that it may contribute to the pathogenesis of choriocarcinoma [60].

### 3.3 Impaired death receptor signalling

Death receptors and ligands of the death receptors are key players in the extrinsic pathway of apoptosis. Other than TNFR1 (also known as DR 1) and Fas (also known as DR2, CD95 or APO-1) mentioned in Section 2.3, examples of death receptors include DR3 (or APO-3), DR4 [or TNF-related apoptosis inducing ligand receptor 1 (TRAIL-1) or APO-2], DR5 (or TRAIL-2), DR 6, ectodysplasin A receptor (EDAR) and nerve growth factor receptor (NGFR) [61]. These receptors posses a death domain and when triggered by a death signal, a number of molecules are attracted to the death domain, resulting in the activation of a signalling cascade. However, death ligands can also bind to decoy death receptors that do not posses a death domain and the latter fail to form signalling complexes and initiate the signalling cascade [61]

Several abnormalities in the death signalling pathways that can lead to evasion of the extrinsic pathway of apoptosis have been identified. Such abnormalities include downregulation of the receptor or impairment of receptor function regardless of the mechanism or type of defects, as well as a reduced level in the death signals, all of which contribute to impaired signalling and hence a reduction of apoptosis. For instance, downregulation of receptor surface expression has been indicated in some studies as a mechanism of acquired drug resistance. A reduced expression of CD95 was found to play a role in treatment-resistant leukaemia [62] or neuroblastoma [63] cells. Reduced membrane expression of death receptors and abnormal expression of decoy receptors have also been reported to play a role in the evasion of the death signalling pathways in various cancers [64]. In a study carried out to examine if changes in death ligand and death receptor expression during different stages of cervical carcinogenesis were related to an imbalance between proliferation and apoptosis, Reesink-Peters *et al *concluded that the loss of Fas and the dysregulation of FasL, DR4, DR5, and tumor necrosis factor-related apoptosis-inducing ligand (TRAIL) in the cervical intraepithelial neoplasia (CIN)-cervical cancer sequence might be responsible for cervical carcinogenesis [65].

## 4. Targeting apoptosis in cancer treatment

Like a double-edged sword, every defect or abnormality along the apoptotic pathways may also be an interesting target of cancer treatment. Drugs or treatment strategies that can restore the apoptotic signalling pathways towards normality have the potential to eliminate cancer cells, which depend on these defects to stay alive. Many recent and important discoveries have opened new doors into potential new classes of anticancer drugs. This Section emphasises on new treatment options targeting some of the apoptotic defects mentioned in Section 3. A summary of these drugs and treatment strategies is given in Table 2.

**Table 2:** Summary of treatment strategies targeting apoptosis

Treatment strategy	Remarks	Author/reference
**Targeting the Bcl-2 family of proteins**		
***Agents that target the Bcl-2 family proteins***	*Oblimersen sodium*	
	Reported to show chemosensitising effects in combined treatment with conventional anticancer drugs in chronic myeloid leukaemia patients and an improvement in survival in these patients	Rai *et al*., 2008 [66], Abou-Nassar and Brown, 2010 [67]
	*Small molecule inhibitors of the Bcl-2 family of proteins*	
	Molecules reported to affect gene or protein expression include sodium butyrate, depsipetide, fenretinide and flavipirodo. Molecules reported to act on the proteins themselves include gossypol, ABT-737, ABT-263, GX15-070 and HA14-1	Kang and Reynold, 2009 [68]
	*BH3 mimetics*	
	ABT-737 reported to inhibit anti-apoptotic proteins such as Bcl-2, Bcl-xL, and Bcl-W and to exhibit cytotoxicity in lymphoma, small cell lung carcinoma cell line and primary patient-derived cells	Oltersdorf *et al*., 2005 [69]
	ATF4, ATF3 and NOXA reported to bind to and inhibit Mcl-1	Albershardt *et al*., 2011 [70]

***Silencing the Bcl family anti-apoptotic proteins/genes***	Bcl-2 specific siRNA reported to specifically inhibit the expression of target gene *in vitro *and *in vivo *with anti-proliferative and pro-apoptotic effects observed in pancreatic carcinoma cells	Ocker *et al*., 2005 [71]
	Silencing Bmi-1 in MCF breast cancer cells reported to downregulate the expression of pAkt and Bcl-2 and to increase sensitivity of these cells to doxorubicin with an increase in apoptotic cells *in vitro *and *in vivo*	Wu *et al*., 2011 [72]

**Targeting p53**		
***p53-based gene therapy***	First report on the use of a wild-type p53 gene containing retroviral vector injected into tumour cells of non-small cell lung carcinoma derived from patients. The use of p53-based gene therapy was reported to be feasible.	Roth *et al*., 1996 [73]
	Introduction of wild type p53 gene reported to sensitise tumour cells of head and neck, colorectal and prostate cancers and glioma to ionising radiation	Chène, 2001 [74]
	Genetically engineered oncolytic adenovirus, ONYX-015 reported to selectively replicate in and lyse tumour cells deficient in p53	Nemunaitis *et al*., 2009 [76]

***p53-based drug therapy***	*Small molecules*	
	Phikan083 reported to bind to and restore mutant p53	Boeckler *et al*., 2008 [77]
	CP-31398 reported to intercalate with DNA and alter and destabilise the DNA-p53 core domain complex, resulting in the restoration of unstable p53 mutants	Rippin *et al*., 2002 [78]
	*Other agents*	
	Nutlins reported to inhibit the MSM2-p53 interaction, stabilise p53 and selectively induce senescence in cancer cells	Shangery and Wang, 2008 [79]
	MI-219 reported to disrupt the MDM2-p53 interaction, resulting in inhibition of cell proliferation, selective apoptosis in tumour cells and complete tumour growth inhibition	Shangery *et al*., 2008 [80]
	Tenovins reported to decrease tumour growth *in vivo*	Lain *et al*., 2008 [81]

***p53-based immunotherapy***	Patients with advanced stage cancer given vaccine containing a recombinant replication-defective adenoviral vector with human wild-type p53 reported to have stable disease	Kuball *et al*., 2002 [82]
	Clinical and p53-specific T cell responses observed in patients given p53 peptide pulsed dendritic cells in a phase I clinical trial	Svane *et al*., 2004 [83]

**Targeting IAPS**		
***Targeting XIAP***	*Antisense approach*	
	Reported to result in an improved *in vivo *tumour control by radiotherapy	Cao *et al*., 2004 [86]
	Concurrent use of antisense oligonucleotides and chemotherapy reported to exhibit enhanced chemotherapeutic activity in lung cancer cells *in vitro *and *in vivo*	Hu *et al*., 2003 [87]
	*siRNA approach*	
	siRNA targeting of XIAP reported to increase radiation sensitivity of human cancer cells independent of TP53 status	Ohnishi *et al*., 2006 [88]
	Targeting XIAP or Survivin by siRNAs sensitised hepatoma cells to death receptor- and chemotherapeutic agent-induced cell death	Yamaguchi *et al*., 2005 [89]

***Targeting Survivin***	*Antisense approach*	
	Transfection of anti-sense Survivin into YUSAC-2 and LOX malignant melanoma cells reported to result in spontaneous apoptosis	Grossman *et al*., 1999 [90]
	Reported to induce apoptosis and sensitise head and neck squamous cell carcinoma cells to chemotherapy	Sharma *et al*., 2005 [91]
	Reported to inhibit growth and proliferation of medullary thyroid carcinoma cells	Du *et al*., 2006 [92]
	*siRNA approach*	
	Reported o downregulate Survivin and diminish radioresistance in pancreatic cancer cells	Kami *et al*., 2005 [93]
	Reported to inhibit proliferation and induce apoptosis in SPCA1 and SH77 human lung adenocarcinoma cells	Liu *et al*., 2011 [94]
	Reported to suppress Survivin expression, inhibit cell proliferation and enhance apoptosis in SKOV3/DDP ovarian cancer cells	Zhang *et al*., 2009 [95]
	Reported to enhance the radiosensitivity of human non-small cell lung cancer cells	Yang *et al*., 2010 [96]

***Other IAP antagonists***	*Small molecules antagonists*	
	Cyclin-dependent kinase inhibitors and Hsp90 inhibitors and gene therapy attempted in targeting Survivin in cancer therapy	Pennati *et al*., 2007 [97]
	Cyclopeptidic Smac mimetics 2 and 3 report to bind to XIAP and cIAP-1/2 and restore the activities of caspases- 9 and 3/-7 inhibited by XIAP	Sun *et al*., 2010 [98]
	SM-164 reported to enhance TRAIL activity by concurrently targeting XIAP and cIAP1	Lu *et al*., 2011 [99]

**Targeting caspases**		
***Caspase-based drug therapy***	Apoptin reported to selectively induce apoptosis in malignant but not normal cells	Rohn *et al*, 2004 [100]
	Small molecules caspase activators reported to lower the activation threshold of caspase or activate caspase, contributing to an increased drug sensitivity of cancer cells	Philchenkov *et al*., 2004 [101]

***Caspase-based gene therapy***	Human caspase-3 gene therapy used in addition to etoposide treatment in an AH130 liver tumour model reported to induce extensive apoptosis and reduce tumour volume	Yamabe *et al*., 1999 [102]
	Gene transfer of constitutively active caspse-3 into HuH7 human hepatoma cells reported to selectively induce apoptosis	Cam *et al*., 2005 [103]
	A recombinant adenovirus carrying immunocaspase 3 reported to exert anticancer effect in hepatocellular carcinoma *in vitro *and *in vivo*	Li *et al*., 2007 [104]

### 4.1 Targeting the Bcl-2 family of proteins

Some potential treatment strategies used in targeting the Bcl-2 family of proteins include the use of therapeutic agents to inhibit the Bcl-2 family of anti-apoptotic proteins or the silencing of the upregulated anti-apoptotic proteins or genes involved.

#### 4.1.1Agents that target the Bcl-2 family of proteins

One good example of these agents is the drug oblimersen sodium, which is a Bcl-2 antisence oblimer, the first agent targeting Bcl-2 to enter clinical trial. The drug has been reported to show chemosensitising effects in combined treatment with conventional anticancer drugs in chronic myeloid leukaemia patients and an improvement in survival in these patients [66,67]. Other examples included in this category are the small molecule inhibitors of the Bcl-2 family of proteins. These can be further divided into: 1) those molecules that affect gene or protein expression and 2) those acting on the proteins themselves. Examples for the first group include sodium butyrate, depsipetide, fenretinide and flavipirodol while the second group includes gossypol, ABT-737, ABT-263, GX15-070 and HA14-1 (reviewed by Kang and Reynold, 2009 [68]).

Some of these small molecules belong to yet another class of drugs called BH3 mimetics, so named because they mimic the binding of the BH3-only proteins to the hydrophobic groove of anti-apoptotic proteins of the Bcl-2 family. One classical example of a BH3 mimetic is ABT-737, which inhibits anti-apoptotic proteins such as Bcl-2, Bcl-xL, and Bcl-W. It was shown to exhibit cytotoxicity in lymphoma, small cell lung carcinoma cell line and primary patient-derived cells and caused regression of established tumours in animal models with a high percentage of cure [69]. Other BH3 mimetics such as ATF4, ATF3 and NOXA have been reported to bind to and inhibit Mcl-1 [70].

#### 4.1.2 Silencing the anti-apoptotic proteins/genes

Rather than using drugs or therapeutic agents to inhibit the anti-apoptotic members of the Bcl-2 family, some studies have demonstrated that by silencing genes coding for the Bcl-2 family of anti-apoptotic proteins, an increase in apoptosis could be achieved. For example, the use of Bcl-2 specific siRNA had been shown to specifically inhibit the expression of target gene *in vitro *and *in vivo *with anti-proliferative and pro-apoptotic effects observed in pancreatic carcinoma cells [71]. On the other hand, Wu *et al *demonstrated that by silencing Bmi-1 in MCF breast cancer cells, the expression of pAkt and Bcl-2 was downregulated, rendering these cells more sensitive to doxorubicin as evidenced by an increase in apoptotic cells *in vitro *and *in vivo *[72].

### 4.2 Targeting p53

Many p53-based strategies have been investigated for cancer treatment. Generally, these can be classified into three broad categories: 1) gene therapy, 2) drug therapy and 3) immunotherapy.

#### 4.2.1 p53-based gene therapy

The first report of p53 gene therapy in 1996 investigated the use of a wild-type p53 gene containing retroviral vector injected into tumour cells of non-small cell lung carcinoma derived from patients and showed that the use of p53-based gene therapy may be feasible [73]. As the use of the p53 gene alone was not enough to eliminate all tumour cells, later studies have investigated the use of p53 gene therapy concurrently with other anticancer strategies. For example, the introduction of wild-type p53 gene has been shown to sensitise tumour cells of head and neck, colorectal and prostate cancers and glioma to ionising radiation [74]. Although a few studies managed to go as far as phase III clinical trials, no final approval from the FDA has been granted so far [75]. Another interesting p53 gene-based strategy was the use of engineered viruses to eliminate p53-deficient cells. One such example is the use of a genetically engineered oncolytic adenovirus, ONYX-015, in which the *E1B-55 kDa *gene has been deleted, giving the virus the ability to selectively replicate in and lyse tumour cells deficient in p53 [76].

#### 4.2.2 p53-based drug therapy

Several drugs have been investigated to target p53 via different mechanisms. One class of drugs are small molecules that can restore mutated p53 back to their wild-type functions. For example, Phikan083, a small molecule and carbazole derivative, has been shown to bind to and restore mutant p53 [77]. Another small molecule, CP-31398, has been found to intercalate with DNA and alter and destabilise the DNA-p53 core domain complex, resulting in the restoration of unstable p53 mutants [78]. Other drugs that have been used to target p53 include the nutlins, MI-219 and the tenovins. Nutlins are analogues of cis-imidazoline, which inhibit the MSM2-p53 interaction, stabilise p53 and selectively induce senescence in cancer cells [79] while MI-219 was reported to disrupt the MDM2-p53 interaction, resulting in inhibition of cell proliferation, selective apoptosis in tumour cells and complete tumour growth inhibition [80]. The tenovins, on the other hand, are small molecule p53 activators, which have been shown to decrease tumour growth *in vivo *[81].

#### 4.2.3 p53-based immunotherapy

Several clinical trials have been carried out using p53 vaccines. In a clinical trial by Kuball *et al*, six patients with advanced-stage cancer were given vaccine containing a recombinant replication-defective adenoviral vector with human wild-type p53. When followed up at 3 months post immunisation, four out of the six patients had stable disease. However, only one patient had stable disease from 7 months onwards [82]. Other than viral-based vaccines, dendritic-cell based vaccines have also been attempted in clinical trials. Svane *et al *tested the use of p53 peptide pulsed dendritic cells in a phase I clinical trial and reported a clinical response in two out of six patients and p53-specific T cell responses in three out of six patients [83]. Other vaccines that have been used including short peptide-based and long peptide-based vaccines (reviewed by Vermeij R *et al*., 2011 [84]).

### 4.3 Targeting the IAPs

#### 4.3.1 Targeting XIAP

When designing novel drugs for cancers, the IAPs are attractive molecular targets. So far, XIAP has been reported to be the most potent inhibitor of apoptosis among all the IAPs. It effectively inhibits the intrinsic as well as extrinsic pathways of apoptosis and it does so by binding and inhibiting upstream caspase-9 and the downstream caspases-3 and -7 [85]. Some novel therapy targeting XIAP include antisense strategies and short interfering RNA (siRNA) molecules. Using the antisense approach, inhibition of XIAP has been reported to result in an improved *in vivo *tumour control by radiotherapy [86]. When used together with anticancer drugs XIAP antisense oligonucleotides have been demonstrated to exhibit enhanced chemotherapeutic activity in lung cancer cells *in vitro *and *in vivo *[87]. On the other hand, Ohnishi *et al *reported that siRNA targeting of XIAP increased radiation sensitivity of human cancer cells independent of TP53 status [88] while Yamaguchi *et al *reported that targeting XIAP or Survivin by siRNAs sensitise hepatoma cells to death receptor- and chemotherapeutic agent-induced cell death [89].

#### 4.3.2 Targeting Survivin

Many studies have investigated various approaches targeting Survivin for cancer intervention. One example is the use of antisense oligonucleotides. Grossman *et al *was among the first to demonstrate the use of the antisense approach in human melanoma cells. It was shown that transfection of anti-sense Survivin into YUSAC-2 and LOX malignant melanoma cells resulted in spontaneous apoptosis in these cells [90]. The anti-sense approach has also been applied in head and neck squamous cell carcinoma and reported to induce apoptosis and sensitise these cells to chemotherapy [91] and in medullary thyroid carcinoma cells, and was found to inhibit growth and proliferation of these cells [92]. Another approach in targeting Survivin is the use of siRNAs, which have been shown to downregulate Survivin and diminish radioresistance in pancreatic cancer cells [93], to inhibit proliferation and induce apoptosis in SPCA1 and SH77 human lung adenocarcinoma cells [94], to suppress Survivin expression, inhibit cell proliferation and enhance apoptosis in SKOV3/DDP ovarian cancer cells [95] as well as to enhance the radiosensitivity of human non-small cell lung cancer cells [96]. Besides, small molecules antagonists of Survivin such as cyclin-dependent kinase inhibitors and Hsp90 inhibitors and gene therapy have also been attempted in targeting Survivin in cancer therapy (reviewed by Pennati *et al*., 2007 [97]).

#### 4.3.3 Other IAP antagonists

Other IAP antagonists include peptidic and non-peptidic small molecules, which act as IAP inhibitors. Two cyclopeptidic Smac mimetics, 2 and 3, which were found to bind to XIAP and cIAP-1/2 and restore the activities of caspases- 9 and 3/-7 inhibited by XIAP were amongst the many examples [98]. On the other hand, SM-164, a non-peptidic IAP inhibitor was reported to strongly enhance TRAIL activity by concurrently targeting XIAP and cIAP1 [99].

### 4.4 Targeting caspases

#### 4.4.1 Caspase-based drug therapy

Several drugs have been designed to synthetically activate caspases. For example, Apoptin is a caspase-inducing agent which was initially derived from chicken anaemia virus and had the ability to selectively induce apoptosis in malignant but not normal cells [100]. Another class of drugs which are activators of caspases are the small molecules caspase activators. These are peptides which contain the arginin-glycine-aspartate motif. They are pro-apoptotic and have the ability to induce auto-activation of procaspase 3 directly. They have also been shown to lower the activation threshold of caspase or activate caspase, contributing to an increase in drug sensitivity of cancer cells [101].

#### 4.4.2 Caspase-based gene therapy

In addition to caspase-based drug therapy, caspase-based gene therapy has been attempted in several studies. For instance, human caspase-3 gene therapy was used in addition to etoposide treatment in an AH130 liver tumour model and was found to induce extensive apoptosis and reduce tumour volume [102] while gene transfer of constitutively active caspse-3 into HuH7 human hepatoma cells selectively induced apoptosis in these cells [103]. Also, a recombinant adenovirus carrying immunocaspase 3 has been shown to exert anti-cancer effects in hepatocellular carcinoma *in vitro *and *in vivo *[104].

### 4.5 Molecules targeting apoptosis in clinical trials

Recently, many new molecules that target apoptosis enter various stages of clinical trials. A search at http://www.clinicaltrials.gov (a registry and results database of federally and privately supported clinical trials conducted in the United States and around the world) returns many results. These molecules target various proteins involved in apoptosis. Many are antagonists of IAPs and molecules that target the Bcl-2 family of proteins. Table 3 summarises ongoing or recently completed clinical trials involving molecules that target apoptosis.

**Table 3:** Ongoing or recently completed clinical trials involving molecules that target apoptosis

Molecule name	Sponsor	Target	Condition	Clinical stage
ABT-263 (in combination with erlotinib or irinotecan)	Abbott	Bcl-2 family of proteins	Solid tumours	Phase I

ABT-263 (in combination with docetaxel)	Abbott	Bcl-2 family of proteins	Solid tumours	Phase I

ABT-263 (in combination with paclitaxel)	Abbott	Bcl-2 family of proteins	Chronic lymphocytic leukaemia	Phase I

ABT-263	Genentech	Bcl-2 family of proteins	Chronic lymphocytic leukaemia	Phase II

AT-101 (Gossypol)	Roswell Park Cancer Institute	Bcl-2 family of proteins	Lymphocytic leukaemia, chronic B-cell leukaemia	Phase I Phase II

AT-406	Ascenta Therapeutics	IAPs	Solid tumours, lymphoma	Phase I

AT-406	Ascenta Therapeutics	IAPs	Acute myelogenous leukaemia	Phase I

ENZ-3042	Therapeutic Advances in Childhood Leukaemia Consortium	IAPs	Acute, childhood and T cell lymphoblastic leukaemia	Phase I

GX15-070MS (Obotoclax)	Children's Oncology Group	Bcl-2 family of proteins	Leukaemia, lymphoma unspecified childhood solid tumour	Phase I

GX15-070MS (Obotoclax)	Arthur G. James Cancer Hospital & Richard J. Solove Research Institute	Bcl-2 family of proteins	Lymphoma	Phase I Phase II

HGS-1029	Human Genome Sciences	IAPs	Advanced solid tumours	Phase I

HGS-1029	Human Genome Sciences	IAPs	Advanced solid tumours	Phase I

LCL-161	Novartis Pharmaceuticals	IAPs	Solid tumours	Phase I

RO5458640	Hoffmann-La Roche	TNF-like weak inducer of apoptosis (TWEAK) ligand	Advanced solid tumours	Phase I

## 5. Conclusions

The abundance of literature suggests that defects along apoptotic pathways play a crucial role in carcinogenesis and that many new treatment strategies targeting apoptosis are feasible and may be used in the treatment of various types of cancer. Some of these discoveries are preclinical while others have already entered clinical trials. Many of these new agents or treatment strategies have also been incorporated into combination therapy involving conventional anticancer drugs in several clinical trials, which may help enhance currently available treatment modalities. However, some puzzling and troubling questions such as whether these treatment strategies induce resistance in tumours and whether they will cause normal cells to die in massive numbers still remain unanswered. This is a true concern if lessons were to be learnt from the conventional anticancer drugs, which wipe out both normal cells and tumour cells and cause brutal side effects and tumour resistance. On the other hand, it would be of clinical benefit, if these molecules that target apoptosis are specifically acting on a single pathway or protein. However, most of the molecules that enter clinical trials act on several targets and these include many inhibitors of the Bcl-family of proteins and some pan-IAP inhibitors. Hence, evidence-based long-term follow ups on patients receiving these new cancer treatments are needed and ongoing research should focus on those strategies that can selectively induce apoptosis in malignant cells and not the normal ones.

## Competing interests

The author declares that there are no competing interests and that this work has not been published or submitted concurrently for publication elsewhere.

## Authors' contributions

RSYW contributed solely to the writing and submission of this work.

## References

[B1] Bauer JH Hefand SL New tricks of an old molecule: lifespan regulation by p53 Aging Cell 2006 5 437 440 10.1111/j.1474-9726.2006.00228.x 16968311 PMC1592233

[B2] Gasco M Shami S Crook T The p53 pathway in breast cancer Breast Cancer Res 2002 4 70 76 11879567 10.1186/bcr426 PMC138723

[B3] Rodrigues NR Rowan A Smith ME Kerr IB Bodmer WF Gannon JV Lane DP p53 mutations in colorectal cancers Proc Natl Acad Sci USA 1990 87 19 7555 7559 10.1073/pnas.87.19.7555 1699228 PMC54786

[B4] Morton JP Timpson P Karim SA Ridgway RA Athineos D Doyle B Jamieson NB Oien KA Lowy AM Brunton VG Frame MC Jeffry Evans TR Sansom OJ Mutant p53 drives metastasis and overcomes growth arrest/senescence in pancreatic cancer PNAS 2010 107 1 246 251 10.1073/pnas.0908428107 20018721 PMC2806749

[B5] Jensen M Engert A Weissinger F Knauf W Kimby E Poynton C Oliff IA Rummel MJ Österborg A Phase I study of a novel pro-apoptotic drug R-etodolac in patients with B-cell chronic lymphocytic leukaemia Invest New Drugs 2008 26 2 139 149 10.1007/s10637-007-9106-z 18094935

[B6] Baritaki S Militello L Malaponte G Spandidos DA Salcedo M Bonavida B The anti-CD20 mAb LFB-R603 interrupts the dysregulated NF-κB/Snail/RKIP/PTEN resistance loop in B-NHL cells: role in sensitization to TRAIL apoptosis Int J Oncol 2011 38 6 1683 1694 21455568 10.3892/ijo.2011.984

[B7] Kerr JF Harmon BV Tomei LD, Cope FO Definition and incidence of apoptosis: an historical perspective Apoptosis: the molecular basis of cell death 1991 3 New York: Cold Spring Harbor Laboratory Press 5 29

[B8] Kerr JFR Wyllie AH Currie AR Apoptosis: a basic biological phenomenon with wide-ranging implications in tissue kinetics Br J Cancer 1972 26 239 257 10.1038/bjc.1972.33 4561027 PMC2008650

[B9] Mohan H Textbook of pathology 2010 5 New Delhi: Jaypee Brothers Medical Publishers 21 60

[B10] Merkle CJ Porth CM, Matfin G Cellular adaptation, injury, and death Pathophysiology: concepts of altered health states 2009 8 Philadelphia: Wolters Kluwer/Lippincott Williams and Wilkins 94 111

[B11] Hacker G The morphology of apoptosis Cell Tissue Res 2000 301 5 17 10.1007/s004410000193 10928277

[B12] Saraste A Pulkki K Morphologic and biochemical hallmarks of apoptosis Cardiovascular Res 2000 45 528 537 10.1016/S0008-6363(99)00384-3 10728374

[B13] Ziegler U Groscurth P Morphological features of cell death News Physiol Sci 2004 19 124 128 10.1152/nips.01519.2004 15143207

[B14] Kroemer G El-Deiry WS Golstein P Peter ME Vaux D Vandenabeele P Zhivotovsky B Blagosklonny MV Malorni W Knight RA Piacentini M Nagata S Melino G Classification of cell death: recommendations of the Nomenclature Committee on Cell Death Cell Death Differ 2005 12 1463 1467 16247491 10.1038/sj.cdd.4401724

[B15] Manjo G Joris I Apoptosis, oncosis, and necrosis. An overview of cell death Am J Pathol 1995 146 3 15 7856735 PMC1870771

[B16] Kumar V Abbas AK Fausto N Aster JC Robins and Cotran: pathologic basis of disease 2010 8 Philadelphia: Saunders Elsevier 25 32

[B17] Hengartner MO Apoptosis: corralling the corpses Cell 2000 104 325 328 10.1016/s0092-8674(01)00219-7 11239389

[B18] Vaux D Silke J Mammalian mitochondrial IAP-binding proteins Biochem Biophy Res Commun 2003 203 449 504 10.1016/s0006-291x(03)00622-3 12729584

[B19] McCarthy NJ Evan GI Methods for detecting and quantifying apoptosis Curr Top Dev Biol 1998 36 259 278 9342533 10.1016/s0070-2153(08)60507-4

[B20] Lavrik IN Golks A Krammer PH Caspases: pharmacological manipulation of cell death J Clin Invest 2005 115 2665 2672 10.1172/JCI26252 16200200 PMC1236692

[B21] Galluzi L Maiuri MC Vitale I Zischka H Castedo M Zitvogel L Kroemer G Cell death modalities: classification and pathophysiological implications Cell Death Differ 2007 14 1237 1266 10.1038/sj.cdd.4402148 17431418

[B22] O'Brien MA Kirby R Apoptosis: a review of pro-apoptotic and anti-apoptotic pathways and dysregulation in disease J Vet Emerg Crit Care 2008 18 6 572 585 10.1111/j.1476-4431.2008.00363.x

[B23] Schneider P Tschopp J Apoptosis induced by death receptors Pharm Acta Helv 2000 74 281 286 10.1016/S0031-6865(99)00038-2 10812970

[B24] Karp G Cell and molecular biology: Concepts and experiments 2008 5 John New Jersey: Wiley and Sons 653 657

[B25] Danial NN Korsmeyer SJ Cell death: critical control points Cell 2004 116 2 205 219 10.1016/S0092-8674(04)00046-7 14744432

[B26] Tsujimoto Y Finger LR Yunis J Nowell PC Croce CM Cloning of the chromosome breakpoint of neoplastic B cells with the t(14; 18) chromosome translocation Science 1984 226 1097 1099 10.1126/science.6093263 6093263

[B27] Reed JC Bcl-2 family proteins: regulators of apoptosis and chemoresistance in haematologic malignancies Semin Haematol 1997 34 9 19 9408956

[B28] Kroemer G Galluzzi L Brenner C Mitochondrial membrane permeabilisation in cell death Physiol Rev 2007 87 1 99 163 10.1152/physrev.00013.2006 17237344

[B29] LaCasse EC Mahoney DJ Cheung HH Plenchette S Baird S Korneluk RG IAP-targeted therapies for cancer Oncogene 2008 27 48 6252 6275 10.1038/onc.2008.302 18931692

[B30] Ghobrial IM Witzig TE Adjei AA Targeting apoptosis pathways in cancer therapy CA Cancer J Clin 2005 55 178 194 10.3322/canjclin.55.3.178 15890640

[B31] Szegezdi E Fitzgerald U Samali Caspase-12 and ER stress mediated apoptosis: the story so far Ann NY Acad Sci 2003 1010 186 194 10.1196/annals.1299.032 15033718

[B32] Hanahan D Weinberg RA The hallmarks of cancer Cell 2000 100 57 70 10.1016/S0092-8674(00)81683-9 10647931

[B33] Gross A McDonnell JM Korsmeyer SJ BCL-2 family members and the mitochondria in apoptosis Genes Dev 1999 13 1899 1911 10.1101/gad.13.15.1899 10444588

[B34] Minn AJ Vélez P Schendel SL Liang H Muchmore SW Fesik SW Fill M Thompson CB Bcl-x(L) forms an ion channel in synthetic lipid membranes Nature 1997 385 6614 353 357 10.1038/385353a0 9002522

[B35] Dewson G Kluc RM Bcl-2 family-regulated apoptosis in health and disease Cell Health and Cytoskeleton 2010 2 9 22

[B36] Raffo AJ Perlman H Chen MW Day ML Streitman JS Buttyan R Overexpression of bcl-2 protects prostate cancer cells from apoptosis in vitro and confers resistance to androgen depletion *in vivo* Cancer Res 1995 55 4438 7671257

[B37] Fulda S Meyer E Debatin KM Inhibition of TRAIL-induced apoptosis by Bcl-2 overexpression Oncogene 2000 21 2283 2294 10.1038/sj.onc.1205258 11948412

[B38] Minn AJ Rudin CM Boise LH Thompson CB Expression of Bcl-XL can confer a multidrug resistance phenotype Blood 1995 86 1903 1910 7655019

[B39] Miquel C Borrini F Grandjouan S Aupérin A Viguier J Velasco V Duvillard P Praz F Sabourin JC Role of bax mutations in apoptosis in colorectal cancers with microsatellite instability Am J Clin Pathol 2005 23 4 562 570 10.1309/JQ2X-3RV3-L8F9-TGYW 15743744

[B40] Goolsby C Paniagua M Tallman M Gartenhaus RB Bcl-2 regulatory pathway is functional in chronic lymphocytic leukaemia Cytometry B Clin Cytom 2005 63 1 36 46 15624202 10.1002/cyto.b.20034

[B41] Pepper C Hoy T Bentley DP Bcl-2/Bax ratios in chronic lymphocytic leukaemia and their correlation with in vitro apoptosis and clinical resistance Br J Cancer 1997 76 7 935 938 10.1038/bjc.1997.487 9328155 PMC2228064

[B42] Levine AJ Momand J Finlay CA The p53 tumour suppressor gene Nature 1991 351 6326 453 456 10.1038/351453a0 2046748

[B43] Bai L Zhu WG p53: structure, function and therapeutic applications J Cancer Mol 2006 2 4 141 153

[B44] Oren M Rotter V Introduction: p53--the first twenty years Cell Mol Life Sci 1999 55 9 11 10.1007/s000180050265 10065147 PMC11146882

[B45] Lane DP p53, guardian of the genome Nature 1992 358 15 16 10.1038/358015a0 1614522

[B46] Avery-Kiejda KA Bowden NA Croft AJ Scurr LL Kairupan CF Ashton KA Talseth-Palmer BA Rizos H Zhang XD Scott RJ Hersey P p53 in human melanoma fails to regulate target genes associated with apoptosis and the cell cycle and may contribute to proliferation BMC Cancer 2011 11 203 10.1186/1471-2407-11-203 21615965 PMC3120805

[B47] Slatter TL Hung N Campbell H Rubio C Mehta R Renshaw P Williams G Wilson M Engelmann A Jeffs A Royds JA Baird MA Braithwaite AW Hyperproliferation, cancer, and inflammation in mice expressing a Δ133p53-like isoform Blood 2011 117 19 5166 5177 10.1182/blood-2010-11-321851 21411755

[B48] Vikhanskaya F Lee MK Mazzoletti M Broggini M Sabapathy K Cancer-derived p53 mutants suppress p53-target gene expression--potential mechanism for gain of function of mutant p53 Nucl Acids Res 2007 35 6 2093 2104 10.1093/nar/gkm099 17344317 PMC1874625

[B49] Vucic D Fairbrother WJ The inhibitor of apoptosis proteins as therapeutic targets in cancer Clin Cancer Res 2007 13 20 5995 6000 10.1158/1078-0432.CCR-07-0729 17947460

[B50] Wei Y Fan T Yu M Inhibitor of apoptosis proteins and apoptosis Acta Biochim Biophys Sin 2008 40 4 278 288 10.1111/j.1745-7270.2008.00407.x 18401525

[B51] Lopes RB Gangeswaran R McNeish IA Wang Y Lemoine NR Expression of the IAP protein family is dysregulated in pancreatic cancer cells and is important for resistance to chemotherapy Int J Cancer 2007 120 11 2344 2352 10.1002/ijc.22554 17311258

[B52] Vucic D Stennicke HR Pisabarro MT Salvesen GS Dixit VM MLIAP, a novel inhibitor of apoptosis that is preferentially expressed in human melanomas Curr Biol 2000 10 1359 1366 10.1016/S0960-9822(00)00781-8 11084335

[B53] Ashhab Y Alian A Polliack A Panet A Ben Yehuda D Two splicing variants of a new inhibitor of apoptosis gene with different biological properties and tissue distribution pattern FEBS Lett 2001 495 56 60 10.1016/S0014-5793(01)02366-3 11322947

[B54] Chen Z Naito M Hori S Mashima T Yamori T Tsuruo T A human IAP-family gene, apollon, expressed in human brain cancer cells Biochem Biophys Res Commun 1999 264 847 854 10.1006/bbrc.1999.1585 10544019

[B55] Small S Keerthivasan G Huang Z Gurbuxani S Crispino JD Overexpression of survivin initiates haematologic malignancies *in vivo* Leukaemia 2010 24 11 1920 1926 10.1038/leu.2010.198 PMC2978276 20882051

[B56] Krepela E Dankova P Moravcikova E Krepelova A Prochazka J Cermak J Schützner J Zatloukal P Benkova K Increased expression of inhibitor of apoptosis proteins, Survivin and XIAP, in non-small cell lung carcinoma Int J Oncol 2009 35 6 1449 1462 19885569 10.3892/ijo_00000464

[B57] Fink SL Cookson BT Apoptosis, pyroptosis, and necrosis: mechanistic description of dead and dying eukaryotic cells Infect Immun 2005 73 4 1907 1916 10.1128/IAI.73.4.1907-1916.2005 15784530 PMC1087413

[B58] Shen XG Wang C Li Y Wang L Zhou B Xu B Jiang X Zhou ZG Sun XF Downregulation of caspase-9 is a frequent event in patients with stage II colorectal cancer and correlates with poor clinical outcome Colorectal Dis 2010 12 12 1213 1218 10.1111/j.1463-1318.2009.02009.x 19604285

[B59] Devarajan E Sahin AA Chen JS Krishnamurthy RR Aggarwal N Brun AM Sapino A Zhang F Sharma D Yang XH Tora AD Mehta K Downregulation of caspase 3 in breast cancer: a possible mechanism for chemoresistance Oncogene 2002 21 57 8843 8851 10.1038/sj.onc.1206044 12483536

[B60] Fong PC Xue WC Ngan HYS Chiu PM Chan KYK Tsao GSW Cheung ANY Caspase activity is downregulated in choriocarcinoma: a cDNA array differential expression study J Clin Pathol 2006 59 2 179 183 10.1136/jcp.2005.028027 16443735 PMC1860314

[B61] Lavrik I Golks A Krammer PH Death receptor signaling J Cell Sci 2005 118 265 267 10.1242/jcs.01610 15654015

[B62] Friesen C Fulda S Debatin KM Deficient activation of the CD95 (APO-1/Fas) system in drug resistant cells Leukaemia 1997 11 11 1833 1841 10.1038/sj.leu.2400827 9369415

[B63] Fulda S Los M Friesen C Debatin KM Chemosensitivity of solid tumour cells in vitro is related to activation of the CD95 system Int J Cancer 1998 76 1 105 114 10.1002/(SICI)1097-0215(19980330)76:1<105::AID-IJC17>3.0.CO;2-B 9533769

[B64] Fulda S Evasion of apoptosis as a cellular stress response in cancer Int J Cell Biol 2010 2010 370835 20182539 10.1155/2010/370835 PMC2825553

[B65] Reesink-Peters N Hougardy BM van den Heuvel FA Ten Hoor KA Hollema H Boezen HM de Vries EG de Jong S van der Zee AG Death receptors and ligands in cervical carcinogenesis: an immunohistochemical study Gynaecol Oncol 2005 96 3 705 713 10.1016/j.ygyno.2004.10.046 15721415

[B66] Rai KR Moore J Wu J Novick SC O'Brien SM Effect of the addition of oblimersen (Bcl-2 antisense) to fludarabine/cyclophosphamide for replased/refractory chronic lymphocytic leukaemia (CLL) on survival in patients who achieve CR/nPR: Five-year follow-up from a randomized phase III study [abstract] J Clin Oncol 2008 26 7008

[B67] Abou-Nassar K Brown JR Novel agents for the treatment of chronic lymphocytic leukaemia Clin Adv Haematol Oncol 2010 8 12 886 895 21326166

[B68] Kang MH Reynolds CP Bcl-2 inhibitors Targeting mitochondrial apoptotic pathways in cancer therapy Clin Cancer Res 2009 15 1126 1132 10.1158/1078-0432.CCR-08-0144 19228717 PMC3182268

[B69] Oltersdorf T Elmore SW Shoemaker AR Armstrong RC Augeri DJ Belli BA Bruncko M Deckwerth TL Dinges J Hajduk PJ Joseph MK Kitada S Korsmeyer SJ Kunzer AR Letai A Li C Mitten MJ Nettesheim DG Ng S Nimmer PM O'Connor JM Oleksijew A Petros AM Reed JC Shen W Tahir SK Thompson CB Tomaselli KJ Wang B Wendt MD Zhang H Fesik SW Rosenberg SH An inhibitor of Bcl-2 family proteins induces regression of solid tumours Nature 2005 435 7042 677 681 10.1038/nature03579 15902208

[B70] Albershardt TC Salerni BL Soderquist RS Bates DJ Pletnev AA Kisselev AF Eastman A Multiple BH3 mimetics antagonize antiapoptotic MCL1 protein by inducing the endoplasmic reticulum stress response and upregulating BH3-only protein NOXA J Biol Chem 2011 286 28 24882 24895 10.1074/jbc.M111.255828 21628457 PMC3137063

[B71] Ocker M Neureiter D Lueders M Zopf S Ganslmayer M Hahn EG Herold C Schuppan D Variants of bcl-2 specific siRNA for silencing antiapoptotic bcl-2 in pancreatic cancer Gut 2005 54 9 1298 1308 10.1136/gut.2004.056192 16099798 PMC1774673

[B72] Wu X Liu X Sengupta J Bu Y Yi F Wang C Shi Y Zhu Y Jiao Q Song F Silencing of Bmi-1 gene by RNA interference enhances sensitivity to doxorubicin in breast cancer cells Indian J Exp Biol 2011 49 2 105 112 21428211

[B73] Roth JA Nguyen D Lawrence DD Kemp BL Carrasco CH Ferson DZ Hong WK Komaki R Lee JJ Nesbitt JC Pisters KM Putnam JB Schea R Shin DM Walsh GL Dolormente MM Han CI Martin FD Yen N Xu K Stephens LC McDonnell TJ Mukhopadhyay T Cai D Retrovirus-mediated wild-type p53 gene transfer to tumuors of patients with lung cancer Nature Medicine 1996 2 9 985 991 10.1038/nm0996-985 8782455

[B74] Chène P p53 as a drug target in cancer therapy Expert Opin Ther Patents 2001 11 6 923 935 10.1517/13543776.11.6.923

[B75] Suzuki K Matusubara H Recent advances in p53 research and cancer treatment J Biomed Biotech 2011 2011 978312 10.1155/2011/978312 PMC3134396 21765642

[B76] John Nemunaitis Ian Ganly Fadlo Khuri James Arseneau Joseph Kuhn Todd McCarty Stephen Landers Phillip Maples Larry Rome Britta Randlev Tony Reid Sam Kaye David Kirn Selective replication and oncolysis in p53 mutant tumors with ONYX-015, an E1B-55kD gene-deleted adenovirus, in patients with advanced head and neck cancer: A phase II trial Cancer Res 2000 60 6359 11103798

[B77] Boeckler FM Joerger AC Jaggi G Rutherford TJ Veprintsev DB Fersht AR Targeted rescue of a destabilised mutant of p53 by an in silico screened drug Proc Natl Acad Sci USA 2008 105 30 10360 10365 10.1073/pnas.0805326105 18650397 PMC2492497

[B78] Rippin TM Bykov VJ Freund SM Selivanova G Wiman KG Fersht A Characterisation of the p53-rescue drug CP-31398 in vitro and in living cells Oncogene 2002 21 14 2119 2129 10.1038/sj.onc.1205362 11948395

[B79] Shangary S Wang S Small-molecule inhibitors of the MDM2-p53 protein-protein interaction to reactivate p53 function: a novel approach for cancer therapy Annu Rev Pharmacol Toxicol 2008 49 223 241 10.1146/annurev.pharmtox.48.113006.094723 PMC2676449 18834305

[B80] Shangary S Qin D McEachern D Liu M Miller RS Qiu S Nikolovska-Coleska Z Ding K Wang G Chen J Bernard D Zhang J Lu Y Gu Q Shah RB Pienta KJ Ling X Kang S Guo M Sun Y Yang D Wang Temporal activation of p53 by a specific MDM2 inhibitor is selectively toxic to tumours and leads to complete tumor growth inhibition Proc Natl Acad Sci USA 2008 105 10 3933 3938 10.1073/pnas.0708917105 18316739 PMC2268798

[B81] Lain S Hollick JJ Campbell J Staples OD Higgins M Aoubala M McCarthy A Appleyard V Murray KE Baker L Thompson A Mathers J Holland SJ Stark MJ Pass G Woods J Lane DP Westwood NJ Discovery, in vivo activity, and mechanism of action of a small-molecule p53 activator Cancer Cell 2008 13 5 454 463 10.1016/j.ccr.2008.03.004 18455128 PMC2742717

[B82] Kuball J Schuler M Antunes Ferreira E Herr W Neumann M Obenauer-Kutner L Westreich L Huber C Wölfel T Theobald M Generating p53-specific cytotoxic T lymphocytes by recombinant adenoviral vector-based vaccination in mice, but not man Gene Ther 2002 9 13 833 843 12080377 10.1038/sj.gt.3301709

[B83] Svane IM Pedersen AE Johnsen HE Nielsen D Kamby C Gaarsdal E Nikolajsen K Buus S Claesson MH Vaccination with p53-peptide-pulsed dendritic cells, of patients with advanced breast cancer: report from a phase I study Cancer Immunol Immunother 2004 53 7 633 641 10.1007/s00262-003-0493-5 14985857 PMC11032806

[B84] Vermeij R Leffers N van der Burg SH Melief CJ Daemen T Nijman HW Immunological and clinical effects of vaccines targeting p53-overexpressing malignancies J Biomed Biotechnol 2011 2011 702146 21541192 10.1155/2011/702146 PMC3085500

[B85] Dai Y Lawrence TS Xu L Overcoming cancer therapy resistance by targeting inhibitors of apoptosis proteins and nuclear factor-kappa B Am J Tranl Res 2009 1 1 1 15 PMC2776288 19966933

[B86] Cao C Mu Y Hallahan DE Lu B XIAP and Survivin as therapeutic targets for radiation sensitisation in preclinical models of lung cancer Oncogene 2004 23 7047 7052 10.1038/sj.onc.1207929 15258565

[B87] Hu Y Cherton-Horvat G Dragowska V Baird S Korneluk RG Durkin JP Mayer LD LaCasse EC Antisense oligonucleotides targeting XIAP induce apoptosis and enhance chemotherapeutic activity against human lung cancer cells *in vitro *and *in vivo* Clin Cancer Res 2003 9 2826 2836 12855663

[B88] Ohnishi K Scuric Z Schiesti RH Okamoto N Takahashi A Ohnishi T siRNA targeting NBS1 or XIAP increases radiation sensitivity of human cancer cells independent of TP53 status Radiat Res 2006 166 454 462 10.1667/RR3606.1 16972754

[B89] Yamaguchi Y Shiraki K Fuke H Inoue T Miyashita K Yamanaka Y Saitou Y Sugimoto K Nakano T Targeting of X-linked inhibitor of apoptosis protein or Survivin by short interfering RNAs sensitises hepatoma cells to TNF-related apoptosis-inducing ligand- and chemotherapeutic agent-induced cell death Oncol Rep 2005 12 1211 1316 16211302

[B90] Grossman D McNiff JM Li F Altieri DC Expression and targeting of the apoptosis inhibitor, Survivin, in human melanoma J Invest Dermatol 1999 113 6 1076 1081 10.1046/j.1523-1747.1999.00776.x 10594755

[B91] Sharma H Sen S Lo ML Mraiggiò Singh N Antisense-mediated downregulation of antiapoptotic proteins induces apoptosis and sensitises head and neck squamous cell carcinoma cells to chemotherapy Cancer Biol Ther 2005 4 720 727 10.4161/cbt.4.7.1783 15917659

[B92] Du ZX Zhang HY Gao DX Wang HQ Li YJ Liu GL Antisurvivin oligonucleotides inhibit growth and induce apoptosis in human medullary thyroid carcinoma cells Exp Mol Med 2006 38 230 240 16819281 10.1038/emm.2006.28

[B93] Kami K Doi R Koizumi M Toyoda E Mori T Ito D Kawaguchi Y Fujimoto K Wada M Miyatake S Imamura M Downregulation of Survivin by siRNA diminishes radioresistance of pancreatic cancer cells Surgery 2005 138 2 299 305 10.1016/j.surg.2005.05.009 16153440

[B94] Liu Q Dong C Li L Sun J Li C Li L Inhibitory effects of the survivin siRNA transfection on human lung adenocarcinoma cells SPCA1 and SH77 Zhongguo Fei Ai Za Zhi 2011 14 1 18 22 21219826 10.3779/j.issn.1009-3419.2011.01.04 PMC5999702

[B95] Zhang X Li N Wang YH Huang Y Xu NZ Wu LY Effects of Survivin siRNA on growth, apoptosis and chemosensitivity of ovarian cancer cells SKOV3/DDP Zhonghua Zhong Liu Za Zhi 2009 31 3 174 177 19615253

[B96] Yang CT Li JM Weng HH Li YC Chen HC Chen MF Adenovirus-mediated transfer of siRNA against Survivin enhances the radiosensitivity of human non-small cell lung cancer cells Cancer Gene Ther 2010 17 120 130 10.1038/cgt.2009.55 19730451

[B97] Pennati M Folini M Zaffaroni N Targeting Survivin in cancer therapy: fulfilled promises and open questions Carcinogenesis 2007 28 6 1133 1139 10.1093/carcin/bgm047 17341657

[B98] Sun H Liu L Lu J Qiu S Yang CY Yi H Wang S Cyclopeptide Smac mimetics as antagonists of IAP proteins Bioorg Med Chem Lett 2010 20 10 3043 3046 10.1016/j.bmcl.2010.03.114 20443226 PMC2997469

[B99] Lu J McEachern D Sun H Bai L Peng Y Qiu S Miller R Liao J Yi H Liu M Bellail A Hao C Sun SY Ting AT Wang S Therapeutic potential and molecular mechanism of a novel, potent, nonpeptide, Smac mimetic SM-164 in combination with TRAIL for cancer treatment Mol Cancer Ther 2011 10 5 902 914 10.1158/1535-7163.MCT-10-0864 21372226 PMC3091962

[B100] Rohn JL Noteborn MH The viral death effector Apoptin reveals tumour-specific processes Apoptosis 2004 9 315 322 15258463 10.1023/b:appt.0000025808.48885.9c

[B101] Philchenkov A Zavelevich M Kroczak TJ Los M Caspases and cancer: mechanisms of inactivation and new treatment modalities Exp Oncol 2004 26 2 82 97 15273659

[B102] Yamabe K Shimizu S Ito T Yoshioka Y Nomura M Narita M Saito I Kanegae Y Matsuda H Cancer gene therapy using a pro-apoptotic gene, caspase-3 Gene Ther 1999 6 12 1952 1959 10.1038/sj.gt.3301041 10637446

[B103] Cam L Boucquey A Coulomb-L'hermine A Weber A Horellou P Gene transfer of constitutively active caspase-3 induces apoptosis in a human hepatoma cell line J Gene Med 2005 7 1 30 38 10.1002/jgm.636 15521050

[B104] Li X Fan R Zou X Gao L Jin H Du R Xia L Fan D Inhibitory effect of recombinant adenovirus carrying immunocaspase-3 on hepatocellular carcinoma Biochem Bioohys Res Commun 2007 358 2 489 494 10.1016/j.bbrc.2007.04.134 17502111

